# Neutrophil degranulation and myocardial infarction

**DOI:** 10.1186/s12964-022-00824-4

**Published:** 2022-04-11

**Authors:** Nan Zhang, Xiahenazi Aiyasiding, Wen-jing Li, Hai-han Liao, Qi-zhu Tang

**Affiliations:** 1 Department of Cardiology, Renmin Hospital of Wuhan University, Wuhan, 430060 People’s Republic of China; 2 Cardiovascular Research Institute of Wuhan University, Wuhan, 430060 People’s Republic of China; 3 Hubei Key Laboratory of Metabolic and Chronic Diseases, Wuhan, 430060 People’s Republic of China

**Keywords:** Myocardial infarction, Neutrophils, Neutrophil degranulation, Myeloperoxidase, Neutrophil elastase, Matrix metalloproteinase, Neutrophil gelatinase-associated lipocalin

## Abstract

Myocardial infarction (MI) is one of the most common cardiac emergencies with high morbidity and is a leading cause of death worldwide. Since MI could develop into a life-threatening emergency and could also seriously affect the life quality of patients, continuous efforts have been made to create an effective strategy to prevent the occurrence of MI and reduce MI-related mortality. Numerous studies have confirmed that neutrophils play important roles in inflammation and innate immunity, which provide the first line of defense against microorganisms by producing inflammatory cytokines and chemokines, releasing reactive oxygen species, and degranulating components of neutrophil cytoplasmic granules to kill pathogens. Recently, researchers reported that neutrophils are closely related to the severity and prognosis of patients with MI, and neutrophil to lymphocyte ratio in post-MI patients had predictive value for major adverse cardiac events. Neutrophils have been increasingly recognized to exert important functions in MI. Especially, granule proteins released by neutrophil degranulation after neutrophil activation have been suggested to involve in the process of MI. This article reviewed the current research progress of neutrophil granules in MI and discusses neutrophil degranulation associated diagnosis and treatment strategies.

**Video abstract**

**Graphical abstract:**

Neutrophils played a crucial role throughout the process of MI, and neutrophil degranulation was the crucial step for the regulative function of neutrophils. Both neutrophils infiltrating and neutrophil degranulation take part in the injury and repair process immediately after the onset of MI. Since different granule subsets (e g. MPO, NE, NGAL, MMP‐8, MMP‐9, cathelicidin, arginase and azurocidin) released from neutrophil degranulation show different effects through diverse mechanisms in MI. In this review, we reviewed the current research progress of neutrophil granules in MI and discusses neutrophil degranulation associated diagnosis and treatment strategies. Myeloperoxidase (MPO); Neutrophil elastase (NE); Neutrophil gelatinase-associated lipocalin (NGAL); Matrix metalloproteinase 8 (MMP‐8); Matrix metalloproteinase 9 (MMP‐9). 
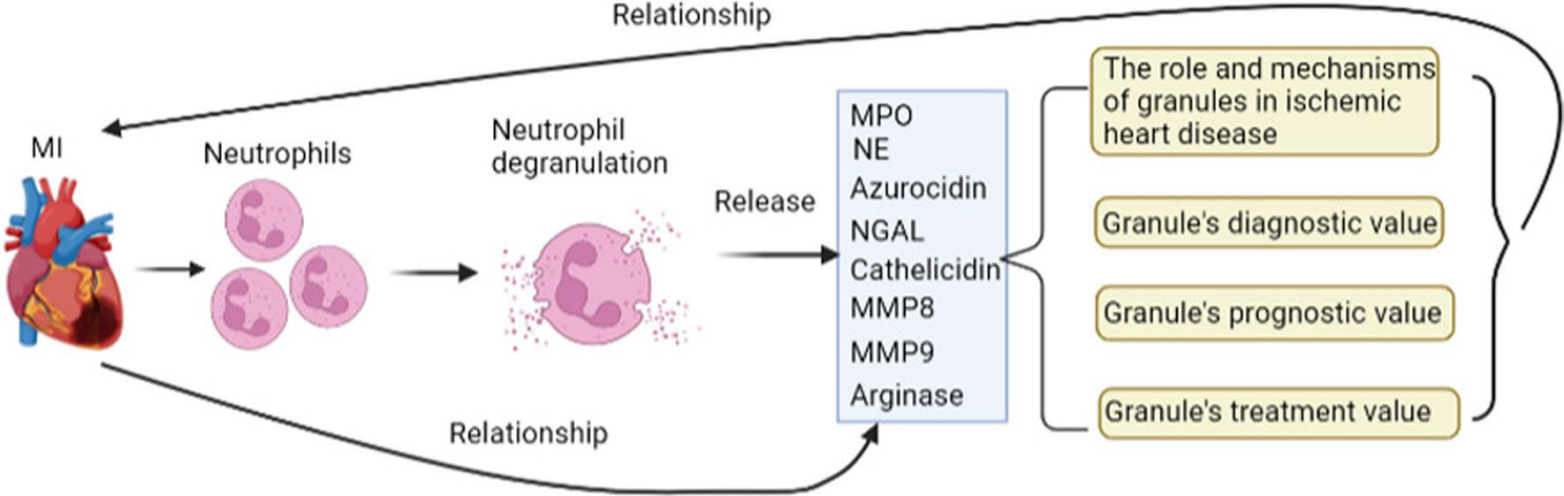

**Supplementary Information:**

The online version contains supplementary material available at 10.1186/s12964-022-00824-4.

## Background

Cardiovascular diseases (CVDs) are still the leading cause of mortality worldwide and responsible for at least one of every three deaths, despite advanced therapeutic interventions for various risk factors related to CVDs [1, 2]. As a typical cardiac emergency, myocardial infarction (MI) is the outcome of ischemic heart disease, including coronary artery stenosis and thrombogenesis, which causes a cascade of cardiac wound healing following myocardial cell necrosis, stimulated inflammation, and leukocyte influx [3]. MI is the leading cause of death for CVDs nowadays and is one of the significant causes of mortality and morbidity. In developing countries, the situation was more serious with an annual growth rate of over 3.6%, which led to 7.4 million deaths per year [4–6]. Thus, it is urgent to find new therapeutic strategies and targets to reduce CVDs-related mortality and morbidity.

Neutrophils are one of the primary inflammatory cells that originate in the bone marrow, mature in response to cytokine stimulation, and then emigrate from the bone marrow into the blood and circulate into tissues [7]. Generally, neutrophils migrate toward the site of inflammation under the guidance of chemokines and cytokines, where they neutralize pathogens by releasing toxic enzymes and proteases through degranulation, producing reactive oxygen species (ROS) by undergoing a respiratory burst, secreting phagosome, and forming Neutrophils Extracellular Traps (NETs) [8–10]. The function of neutrophils is highly dependent on the composition of their cytoplasmic granules. Cytoplasmic granules mobilize to fuse with the plasma membrane (exocytosis), endocytic vacuoles (endocytosis), or other granules that play important roles in inflammation-associated diseases [11]. Neutrophil degranulation releases proteases to degrade the extracellular matrix (ECM) and facilitate leukocyte infiltration. There are four major types of cytoplasmic granules of neutrophils, namely primary, secondary, tertiary, and secretory granules, the contents of which are synthesized at different differentiation stages [12]. The primary granules are also called azurophil granules, the largest and earliest formed granules, containing myeloperoxidase (MPO), neutrophil elastase (NE), cathepsin G, serine proteases, azurocidin, α-defensins, lysozyme, most proteolytic and bactericidal/permeability-increasing proteins [13]. Primary granules can increase the expression of CD63 on the cell surface and promote the release of MPO, NE and arginase-1 (ARG1). The secondary granules are also called specific granules, which contain lactoferrin, neutrophil gelatinase-associated lipocalin (NGAL, lipocalin-2), cathelicidin, and lysozyme. Secondary granules could increase the expression of CD66b/CD15 on the cellular surface and promote the release of NGAL and S100A8/A9. The tertiary granules, also called gelatinase granules, are enriched in matrix metalloproteinase-9 (MMP-9), MMP-8, and a few microbicidal materials. Tertiary granules could promote the expression of CD11b/CD18 on the cellular surface and enhance the release of MMP-9, heparin enzyme, and ARG1. Secretory granules consist primarily of complement receptor 1, plasma protein albumin, CD13 (aminopeptidase N), CD14, and CD16 (Fc gamma receptor III) [13]. Neutrophil granule subsets transfer into the tissue in a certain order: secretory > tertiary granules > secondary granules > primary granules [14] (Table 1).

**Table 1:** List of different types of granules, with their components and characteristics

Types	Components	Release	Characteristics
Primary granules (azurophilic)	MPO NE Cathepsin G Proteinase 3 Azurocidin α-defensins Lysozyme Proteolytic proteins BPI	MPO NE ARG1	1. Earliest formed 2. Last degranulated 3. The size is largest
Secondary granules (specific)	Lactoferrin NGAL Cathelicidin Lysozyme Alkaline phosphatase, NADPH oxidase Collagenase	NGAL S100A8/A9	1. Second formed 2. Discharged before primary granules 3. Smaller than azurophilic granules
Tertiary granules (gelatinase)	Cathepsin, Gelatinase MMP-9 MMP-8 Microbicidal materials	MMP-9, Heparin enzyme ARG1	1. Third formed 2. Discharged followed by secretory granules 3. Smaller than secondary granules
Secretory granules	CR1, Plasma protein albumin CD13 CD14, CD16		1. Last formed 2. Discharged first 3. The size is smallest

*MPO* myeloperoxidase, *NE* neutrophil elastase, *ARG1* arginase-1, *BPI* bactericidal/permeability-increasing protein, *NGAL* neutrophil gelatinase-associated lipocalin, *NADPH* nicotinamide adenine dinucleotide phosphate, *S100A8/A9* S100 calcium binding protein A8/A9, *MMP* matrix metalloprotease, *CR1* complement receptor 1, *CD13* aminopeptidase N, *CD16* Fc gamma receptor III

Studies have reported that neutrophils played a crucial role throughout the process of MI. Within hours after MI, a large number of neutrophils are firstly recruited to the infarct area. Neutrophils infiltrate and interact with necrosis, and apoptosis cells to propagate inflammation, which initiates an acute inflammatory response to engulf dead cells and tissue debris for facilitating reparative phase transformation [14]. During the process of MI, the number of neutrophils peaks at days 1–3, and starts to decline from day 5, which declines to baseline or lower levels at day 7 post-MI [14]. In addition, cardiac neutrophils undergo polarization, continual and distinct proteomic evolution over the first week of MI. Excessive neutrophil infiltration or delayed regression exacerbates tissue injury because of excessive production and accumulation of inflammatory mediators and proteinases [15]. Thus, modest neutrophil recruitment is essential for cardiac healing after MI [16], but excessive infiltration and delayed regression of neutrophils is destructive for cardiac healing.

During the process of neutrophil recruitment in MI, various reports have been reported that neutrophil degranulation was the crucial step for the regulative function of neutrophils during MI. By conducting systematic analysis via aggregating five coronary heart disease microarray datasets from the GEO series, Shi et al. [17] conclude that neutrophil degranulation was one of the most critical processes associated with coronary heart disease. Hoenderdos et al. [18] demonstrated that hypoxia enhanced neutrophil degranulation, which led to the shift of harmful proteins and proteases into the extracellular milieu resulted in aggravated tissue injuries. Maximilian Mauler found that serotonin secreted from platelets promote neutrophil degranulation with CD11b externalization and boost myeloperoxidase (MPO) and hydrogen peroxide (H_2_O_2_) secretion, which finally aggravated myocardial ischemia/reperfusion injury [19]. Similarly, another study found that serotonin-induced exocytosis of neutrophil granules increased the surface expression of the β2-integrin CD11b, which mediates cell adhesion to platelets and endothelium and releases MPO and H_2_O_2_, all of which hampered the healing process after MI [20].

Neutrophil degranulation was activated immediately after MI on the first day, evidenced by the production of calgranulin B (S100A9), activin A, histone H1.2, and fibrinogen [21]. Increased MMP-8 and MMP-9 could further demonstrate the occurrence of neutrophils degranulation at day 1 after MI onset [21]. MMP-8 and MMP-9 are secreted into the ECM by neutrophil gelatinase granules to degrade ECM and promote inflammatory signaling [21]. After MI onset 3 days, cathepsin D and erythropoietin receptor (EPO-R) reached their highest peak accompanied by the initial activation of inflammation resolution signaling. Cathepsins were secreted during neutrophils degranulation, especially from ficolin-1 rich granules [21]. On the fifth day of MI, the most increased secretions were cathepsins D and B, calgranulin b, α-synuclein, fibrinogen, and fibronectin [21]. On the seventh day after MI, the numbers of neutrophils are significantly decreased, still, the expression of cathepsin B, fibrinogen, and fibronectin remained to keep at a relatively high level, and the galectin-3 and S100A4 were also significantly up-regulated in the infarct area [21]. These cytokines indicated the transformation of neutrophils from N1 to N2, which could contribute to scar formation and promote infarct area repairment by stimulating ECM reorganization [22]. The above description could conclude that neutrophil infiltrating and neutrophil degranulation take part in the injury and repair process immediately after the onset of MI. Since different granule subsets released from neutrophil degranulation show different effects through diverse mechanisms in MI, in this review, we reviewed the roles of granule subsets in MI in detail.

## Myeloperoxidase (MPO)

Myeloperoxidase (MPO), a heme enzyme, is mainly derived from granulocytes and monocytes and was stored in the primary azurophilic granules of the neutrophils. In response to inflammatory stimuli, MPO is released from the primary azurophilic granules and uses hydrogen peroxide to catalyze the oxidation of halide ions to hypohalous acids, which could damage or disrupt structures of amino acids, and some macromolecules resulted in the dysfunction of their normal biology [23]. MPO showed protective effects against microbial infections; however, it has been demonstrated to be a critical pro-inflammatory enzyme that caused tissue injuries in cardiovascular, neurological, and rheumatological diseases [24]. In recent years, MPO has been attracting considerable interest as a candidate biomarker for risk stratification of CAD progress and atherosclerotic plaque instability [25, 26].

### MPO-associated mechanisms in ischemic heart disease

MPO is one of the main functional proteins of neutrophils, accounting for about 5% of the dry weight of the neutrophils. It is stored in the azurophilic granules, and about 30% of MPO can be released extracellularly by degranulation or binding to the extracellular trap of the neutrophils. MPO is also found in monocytes, at a much lower concentration than in neutrophils [27, 28]. MPO is a 146 KDa homodimer protein consisting of functionally independent monomer units linked by a single disulfide bond at Cys153 [29]. MPO is linked to heme by three covalent bonds and its activity is dependent on Asn421, which is attached to the proximal end of the heme group. As a hydrogen bond receptor, Asn421 promotes Fe^3+^/Fe^2+^ reduction, which is requisite for the compound I formation. His95 is located distally to heme and accepts protons of H_2_O_2_, triggering the formation of compound I [30]. In the reaction cycle between MPO and complex I, HOCl is the main MPO-derived oxidant. Under physiological conditions, HOCl activity exceeds that of H_2_O_2_, peroxynitrite, and hydroperoxides. When HOCl is overproduced and accumulated, it could destroy the redox pathway and disturb cellular homeostasis by reactivating with mercaptan and thioether [31]. In addition, HOCl could disrupt the biological functions of proteins by interacting with Cys-rich active sites. An investigation has indicated that HOCl could inactivate endothelial nitric oxide synthase (eNOS), creatine kinase, and glyceraldehyde 3-phosphate dehydrogenase by binding their Cys sites [25]. Moreover, HOCl could induce MMP-7 overproduction by oxidizing key Cys residues in the cysteine switching domain of MMP-7 [32].

MPO is involved in low-density lipoprotein (LDL) oxidation pathways, including free radical 1e-oxidation and non-free radical 2e-oxidation [33]. HOCl chlorinates electron-rich substrates on apolipoprotein B-100, such as Lys and Tyr residues, forming MPO-specific 3-chlorotyrosine [34]. In addition, the MPO/HOCl system can produce a series of secondary oxidation products, such as tyrosine radicals, p-hydroxyphenylacetaldehyde, and highly reactivated unsaturated aldehyde-glyceraldehyde, 2-hydroxypropyl, and acrolein. These secondary oxidation products can participate in the oxidation reaction to induce a high intake of LDL [33]. MPO could also oxidize apolipoprotein A-I (Apo A-I), the major high-density lipoprotein protein (HDL). HDL has been suggested to retard atherosclerosis by promoting cholesterol efflux. However, MPO-mediated Apo A-I oxidation could impair cholesterol efflux and fail to activate the cholesterol acyltransferase of lecithin, which could transform free cholesterol to cholesteryl ester to promote HDL maturation [35]. Because MPO could cause HDL particles dysfunction, which is very critical for MI incidence and prognosis, it has been discussed that MPO-modified HDL is causally linked to incidence and prognosis of MI [36]. Thus, MPO might be a potential target to stratify MI patients and highlight clinical benefits for MI patients. However, it needs more prospective studies to further establish a direct causal link to the incidence and prognosis of MI. MPO/HOCl system could also limit the bioavailability of nitric oxide (NO) caused endothelial dysfunction [25]. MPO and its reaction products may disrupt NO formation and bioavailability in the following ways: Firstly, HOCl could chlorinate arginine, the endothelial nitric oxide synthase (eNOS). Chlorided arginine limits the bioavailability of arginine resulted in inhibited eNOS activity. Secondly, HOCl can directly oxidize eNOS resulted in the decoupling of synthase. Thirdly, MPO/H_2_O_2_/NO_2_ system mediated lipoprotein modification may lead to the separation of eNOS from the plasma membrane of endothelial cells resulting in decreased eNOS expression [25].

MPO could also regulate post-MI-associated cardiac remodeling by regulating MMPs. MMP-9 and MPO could be released from tertiary and primary granules during neutrophil activation and degranulation to promote inflammatory disorders [37]. MPO used H2O2 to generate HOCl, which could oxidize the thiol residue of the pro-MMP-7. The oxidized thiol could promote autolytic cleavage of pro-MMP-7 resulted in MMP activation. Activated MMP7 had been demonstrated to cause plaque rupture in the artery wall and exacerbate pathological cardiac remodeling after MI [32]. Mollenhauer, M et al. also demonstrated that MPO could mediate MMP-7 activation resulted in accumulated myofibroblasts and increased post ischemic fibrosis. However, MPO knockout significantly inhibited fibroblast-to myofibroblast transdifferentiation resulted in alleviated pathological cardiac remodeling [38].

Tissue inhibitors of metalloproteinases (TIMPs) could inhibit MMP activity, an imbalance between the proteolytic activity of TIMPs and MMPs is a critical factor for maintaining a balance of cellular matrix and protesting against pathological cardiac fibrosis [39]. However, HOCl generated by the MPO-H2O2-chloride system could oxidize N-terminal cysteine of TIMP-1, which markedly prevents TIMP-1 from inhibiting MMPS [39]. This study suggested that MPO mediated HOCl production could impair TIMP-1 activity during ischemia-associated inflammation response, which exacerbated pathological cardiac fibrosis.

**Fig. 1:**
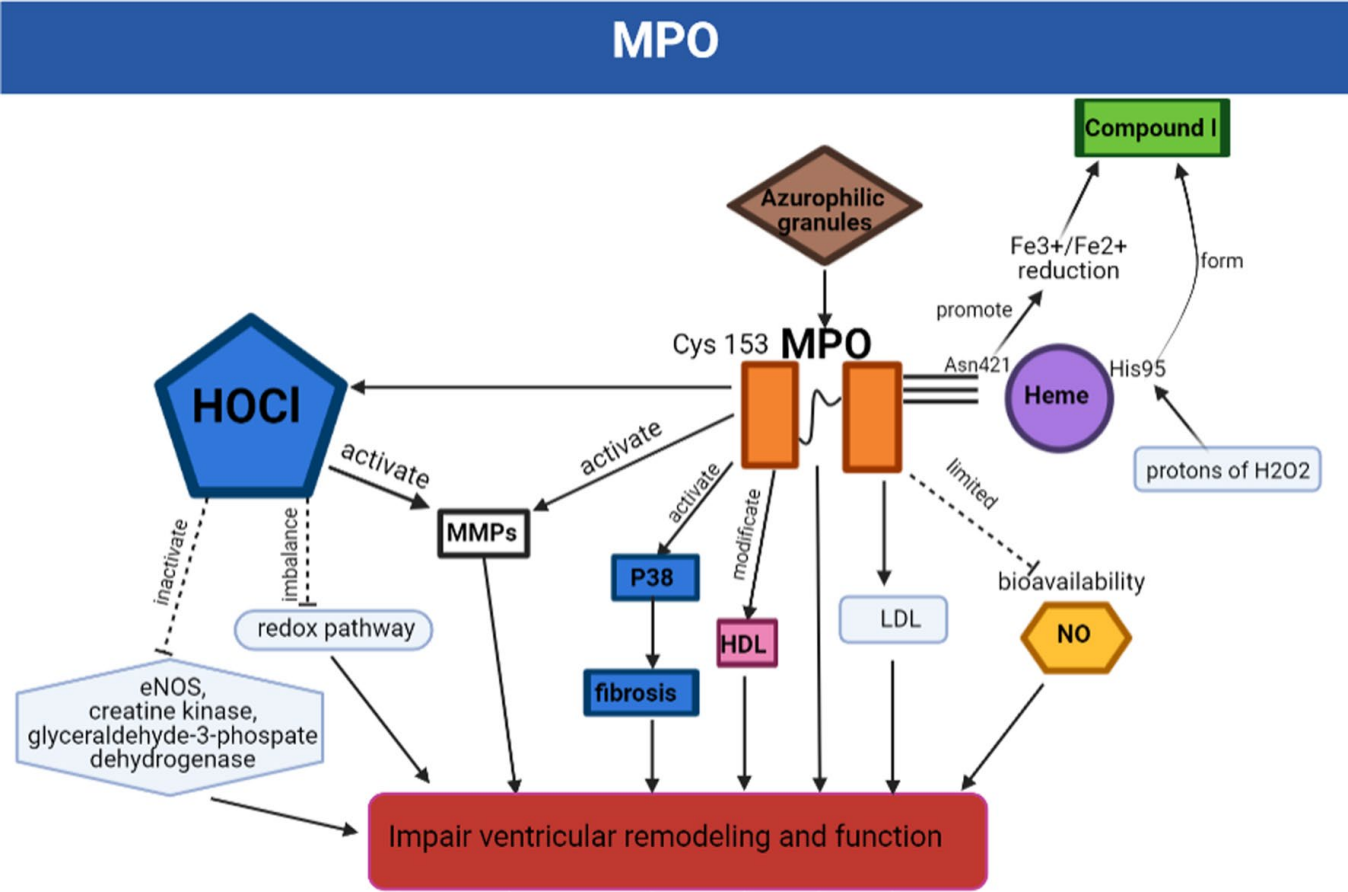
MPO-associated mechanisms in ischemic heart disease. MPO is released by azurophilic granules. MPO is linked to heme by three covalent bonds, and its activity is dependent on Asn421, Asn421 promotes Fe^3+^/Fe^2+^ reduction and contributes to the formation of compound I. One of the main MPO-derived oxidants is HOCl, which affects cell homeostasis by many pathways. In addition, MPO can impair ventricular remodeling and function by affecting MMPs, P38, HDL, LDH, or NO-related signaling pathways. *Abridgment MPO* myeloperoxidase, *Cys* cysteine, *Asn* asparagines, *NO* nitric oxide, *H*_*2*_*O*_*2*_ hydrogen peroxide, *LDL* low-density lipoprotein, *eNOS* endothelial nitric oxide synthase, *HOCl* hypochlorous acidm, *MMPs* matrix metalloproteinases proteins, *HDL* high-density lipoprotein

In summary, MPO has been demonstrated to be significantly over-generated in neutrophils and monocytes in ischemia-associated cardiac injuries. MPO-mediated oxidative stress might play a key role in ischemia/reperfusion-related injuries. Besides MPO-mediated direct oxidative stress injuries, MPO could also modify the cellular component resulted in destroyed molecular function. MPO-mediated lipoprotein modification, and dysfunction might impair vascular reactivity, accelerate atherosclerosis and enhance atherosclerotic plaque instability. MPO-mediated MMPs or TIMPs modify, and dysfunction might destroy the balance between MMPs and TIMPs resulted in exaggerated myocardial remodeling after MI. Thus, inhibiting MPO activity might be a potential strategy for alleviating ischemia/reperfusion-related injuries and limiting MI-associated adverse cardiac remodeling (Fig. 1).

### Diagnostic value of MPO

Some studies have suggested that MPO might predict AMI and provide incremental information for discriminating ACS from other etiologies associated with chest pain.

Omran et al. [40] presented that MPO was a more efficient marker compared to creatine kinase (CK) MB and Troponin I (cTn I) to discriminate AMI from non-coronary chest pain patients, stable angina patients, and unstable angina patients within 0–6 h after the onset of AMI. A combination of MPO, CK-MB, and Tn I could discriminate 91% of the AMI patients as high as a specificity of 76% [40]. MPO and Tn I were markedly associated with adverse cardiovascular events during hospitalization in a prospective cohort study including 11 patients with detected ACS within 24 h [41]. MPO as a valid test detection of MI yielded a specificity of 0.85 [41]. In MI patients, plasma MPO levels were increased by sevenfold [42]. During 1203 days of follow-up among 185 patients, higher MPO prospectively forecasts the outcome of major adverse cardiovascular events (MACE) [42]. In a study of 274 consecutive chest pain patients, MPO levels increased in patients finally diagnosed with AMI even when Tn I exhibited a negative result at an early stage [43]. Although Tn I yielded a higher negative predictive value (NPV) (91.7%, 95% CI 89.5–94.0) and a higher sensitivity (85.9%, 95% CI 82.3–89.5) for diagnosing AMI than that of MPO (NPV of 85.5%, 95% CI 82.6–88.4 and sensitivity of 80%, 95% CI 75.8–84.2) in all patients [43], MPO yielded a NPV of 95.6% (95% CI 94.0–97.3) and a sensitivity of 95.8% (95% CI 93.7–97.9) in AMI patients with a symptom onset of less than 2 h, which is more efficient than that of TnI with a NPV of 73.3% (95% CI 69.8–76.9) and a sensitivity of 50% (95% CI 44.8.7–55.2) [43]. In 432 consecutive patients admitted to the emergency department with ACS, MPO yielded a sensitivity of 82.1% and specificity of 37.2% for forecasting MACE compared to a sensitivity of 60.7% and specificity of 61.4% for the highly sensitive cTnI [44]. Moreover, MPO exhibited a strong prognosis value for MACE in serial sensitive cTnI negative patients [44]. These studies demonstrated that MPO might be a more efficient marker for diagnosing AMI and MACE than the highly sensitive TnI; however, these studies were the small size of patients and lacked a cut-off value for MPO. To solve these defects and deficiencies, Rudolph et al. [45] collected 1880 consecutive patients admitted to the emergency department by the onset of chest pain. They also defined the cut-off for MPO in 5000 population-based subjects [45]. Their study demonstrated that MPO was inferior to the highly sensitive TnI in predicting AMI at 3 h and 6 h after admission of patients with chest pain [45]. MPO only yielded a sensitivity of 73.5% and specificity of 45.5% compared with a sensitivity of 90.7% and specificity of 90.2% in the highly sensitive TnI [45]. So, it remained controversial whether MPO could provide incremental information in predicting AMI and discriminating AMI from patients with chest pain. Nevertheless, it is undoubtedly that MPO is significantly up-regulated in ACS, so it is interesting and meaningful to investigate further the diagnostic role of MPO in ACS comparing TnI and some other prognostic markers. It remained to need well-designed prospective large-sample studies to clarify whether MPO was an excellent diagnostic marker for detecting ACS at the early stage.

### Prognostic value of MPO

Several studies have reported that the MPO was significantly associated with adverse cardiovascular events in AMI patients during hospitalization [41]. Higher MPO predicted worse cardiac outcomes and lower ejection fraction [46, 47], indicating higher long-term mortality [48]. Koch et al. [49] presented that greater than 306.3 pmol/L of MPO measured 24 h after the onset of symptoms was an independent predictor of 6-month mortality and major adverse cardiac events in patients with suspected MI. Rudolph et al. [45] showed that MPO was a predictive marker of increased risk of adverse events at 30 days and 6 months in patients admitted with ACS. Furthermore, plasma MPO levels are significantly related to plaque erosion in patients with ST-segment elevation MI (STEMI) [50]. Stamboul et al. [51] found that in patients with AMI, a high MPO level in the culprit artery was associated with more severe microvascular obstruction (MO) and greater infarct size (IS). In the first week after AMI, the extent of the MO was significantly greater in the high-MPO group, together with greater infarct size, and a trend towards a lower left ventricular ejection fraction [51]. Cardiac magnetic resonance also demonstrated that higher MPO in the culprit artery indicated an exacerbated cardiac remodeling and infarct area at 6 months [51].

However, analysis from a total of 597 hospitalized ACS patients revealed that the level of plasma MPO was significantly higher in STEMI patients than in NSTE-ACS patients. But, MPO could not predict the short-term or long-term outcomes in patients with ACS [52]. Whether MPO has an independent prognostic value or predicts the outcomes in patients with MI deserves further research. The different research results regarding the importance of MPO in the diagnosis and prognosis of MI may partly relate to the lack of a unified method for quantification. Thus, developing a reliable way to quantify the activity and concentration of MPO may be necessary, which is a recent direction for some researches [53].

### Treatment value of MPO

MPO inhibition has been demonstrated to improve ischemia associated cardiac remodeling in animal experiments. In mouse MI models, administration of PF-1355 (an oral MPO inhibitor) for 7 days decreased inflammatory cell infiltration and attenuated left ventricular dilation [20]. Both the cardiac function and remodeling were significantly improved after 21 days of constant treatment [20]. This study implied that MPO inhibition in acute ischemia patients might preserve cardiac function and attenuate pathological cardiac remodeling.

## Neutrophil elastase (NE)

Neutrophil elastase (NE), mainly existed in primary granules, was significantly associated with endotoxemia-induced myocardial injuries. NE inhibition could be a helpful strategy in treating endotoxemia [54]. NE is a serine protease rapidly released extracellularly from azurophilic granules upon neutrophil activation. It acts on a wide range of substrates, including extracellular matrix components, proenzymes, adhesion molecules, signal receptors, and cytokines [55, 56]. The secreted serine proteinases from neutrophils could kill invading pathogens and resolve the inflammation caused by bacterial infection. [57]. However, the constant secretion of NE could cause tissue destruction, NE inhibition could be a useful strategy in attenuating endotoxemia-associated mouse cardiac injuries [54]. An extensive body of literature documents the involvement of NE in tissue destruction and inflammation in arthritis, respiratory diseases, and cardiovascular insults, including I/R injury [58].

### The roles and mechanisms of NE in ischemic heart disease

Elastin, collagen, and fibrinogen are all degraded by NE, which leads to cardiac damage after a heart attack. By inducing IL-6 release through a nitric oxide-dependent mechanism, NE impairs cardiac contractility [59]. NE can cleave and activate pro-MMP-9, showing that PMN-derived molecules interact [60]. Reports have been reported that patients with acute MI have higher plasma concentrations of NE. Furthermore, there is a selective and non-redundant role for NE in I/R-induced neutrophil migration through venular walls as mediated by the remodeling of the venular basement membrane. NE deletion suppressed neutrophil migration into ischemic regions in NE knockout mice compared to wild type mice in I/R injury [61]. The protective effect was associated with reducing neutrophil activation and vascular leakage [61]. NE reached its peak on day1 in the infarcted mouse hearts [62]. NE deficiency decreased mouse mortality, increased cardiac function, and reduced fibrosis in the non-infarcted myocardium [62]. The underlying mechanisms might be associated with reducing cardiomyocyte apoptosis via upregulating insulin/Akt signaling post-MI [62].

### NE’s treatment value

Because NE mediated malignant remodeling in ischemic cardiomyopathy, scientists have done their best to look for a NE inhibitor for treating ischemia-associated injuries. Sivelestat, an inhibitor, has improved survival and preserved cardiac function in mouse MI models [62]. Therapeutic inhibition of NE has demonstrated promising results in preclinical models of inflammatory lung and bowel disease and I/R injury [63]. In MI, pharmacological targeting of NE has shown promising anti-inflammatory efficacy in several experimental and clinical settings of I/R injury and is considered a plausible clinical strategy for organ care [61]. Sivelestat, a NE inhibitor, improved global ischemia-induced myocardial damage, and coronary endothelial dysfunction, ameliorated myocardial contractile dysfunction due to myocardial stunning by inhibiting neutrophil-derived elastase, and attenuated myocardial injury after cardioplegic arrest in rat hearts. This cardio-protective effect was achieved even when Sivelestat was administered during early reperfusion in swine [64, 65]. SSR69071, an elastase inhibitor, has been found to diminish the size of infarcts following ischemia–reperfusion injury by inhibiting NE [66]. In pigs, a specific NE inhibitor prevents myocardium stunning following I/R [64]. In patients with MI, the effects of NE inhibitor deserve more effort and clinic researches.

## Azurocidin

Azurocidin or heparin-binding protein, also known as cationic antimicrobial peptide 37, is a 37 kDa antimicrobial protein contained in the chymosin-like protease serprocidin subgroup and stored in both azurophilic granules and secretory vesicles of the neutrophils [67]. Azurocidin is a protein with multiple biological functions, including exerting antibacterial activity, inducing monocyte recruitment to inflammatory sites, and enhancing macrophage phagocytosis [68]. Azurocidin could induce calcium dependent cytoskeletal rearrangement, and increased macromolecular efflux in endothelial-cell and micro-vessels resulted in enhanced endothelial cell permeability and neutrophil trafficking in inflammation [69, 70]. Targeting azurocidin in the inflammatory response in ischemic heart disease might provide a new strategy for inhibiting endothelial barrier dysfunction caused by neutrophil activation.

### The roles and mechanisms of Azurocidin in ischemic heart disease

Azurocidin levels were significantly upregulated in patients with ST segment elevation myocardial infarction (STEMI) [71]. In an analysis of receiver-operating characteristic curve analysis, an azurocidin cut-off level of > 11.46 ng/mL showed 74% sensitivity and 58% specificity in forecasting STEMI [71]. In the analysis of multivariate linear regression analysis, azurocidin was closely associated with the thrombolysis in myocardial infarction (TIMI) score [71]. This study indicated that azurocidin might be necessary for patients with STEMI [71]. In a randomized controlled trial, simvastatin treatment could significantly reduce plasma azurocidin levels and improve the outcome of patients with acute lung injury in the intensive care unit [67]. This study implied that STEMI patients benefit from statin treatment partly from preventing azurocidin associated inflammatory response.

## Neutrophil gelatinase-associated lipocalin (NGAL)

Neutrophil gelatinase-associated lipocalin (NGAL) is a 25 KDa small glycoprotein secreted by neutrophils, belonging to the lipocalin superfamily [72]. Neutrophils are the primary source of plasma NGAL [73]. However, it was also expressed in many other organs and cells, such as the kidney, endothelial, liver, smooth muscle cells (SMC), cardiomyocytes, cardiac fibroblast, neurons, and various immune cell populations [74]. NGAL has been demonstrated to have multiple functions: NGAL could regulate iron homeostasis by binding to siderophores for impacting oxidative stress, inflammation, apoptosis, and fibrosis [74]. NGAL could promote immune cells migration and invasion [74]. NGAL could promote the differentiation and proliferation of vascular SMCs, cardiac fibroblasts, and some other type cells by acting as a growth factor [74]. Increased evidence suggests that NGAL may involve inflammatory reactions and early acute injury in cardiovascular diseases [75]. NGAL protein levels were significantly upregulated in the left ventricular at 7 days post myocardial infarction [76]. NAGL could regulate the enzymatic activity of matrix metalloproteinase-9 (MMP-9) and promote the formation of a complex between NGAL and MMP-9, which could exacerbate the progression of the atherothrombotic disease [75]. Interestingly, plasma NGAL indicated the inflammatory response in STEMI patients with regular estimated glomerular filtration rate (eGFR); however, plasma NGAL might reflect kidney function in STEMI patients with reduced eGFR [77]. This study implied that we should not ignore kidney function by using NGAL to evaluate inflammatory levels in STMI patients.

### The roles and mechanisms of NGAL in ischemic heart disease

**Fig. 2:**
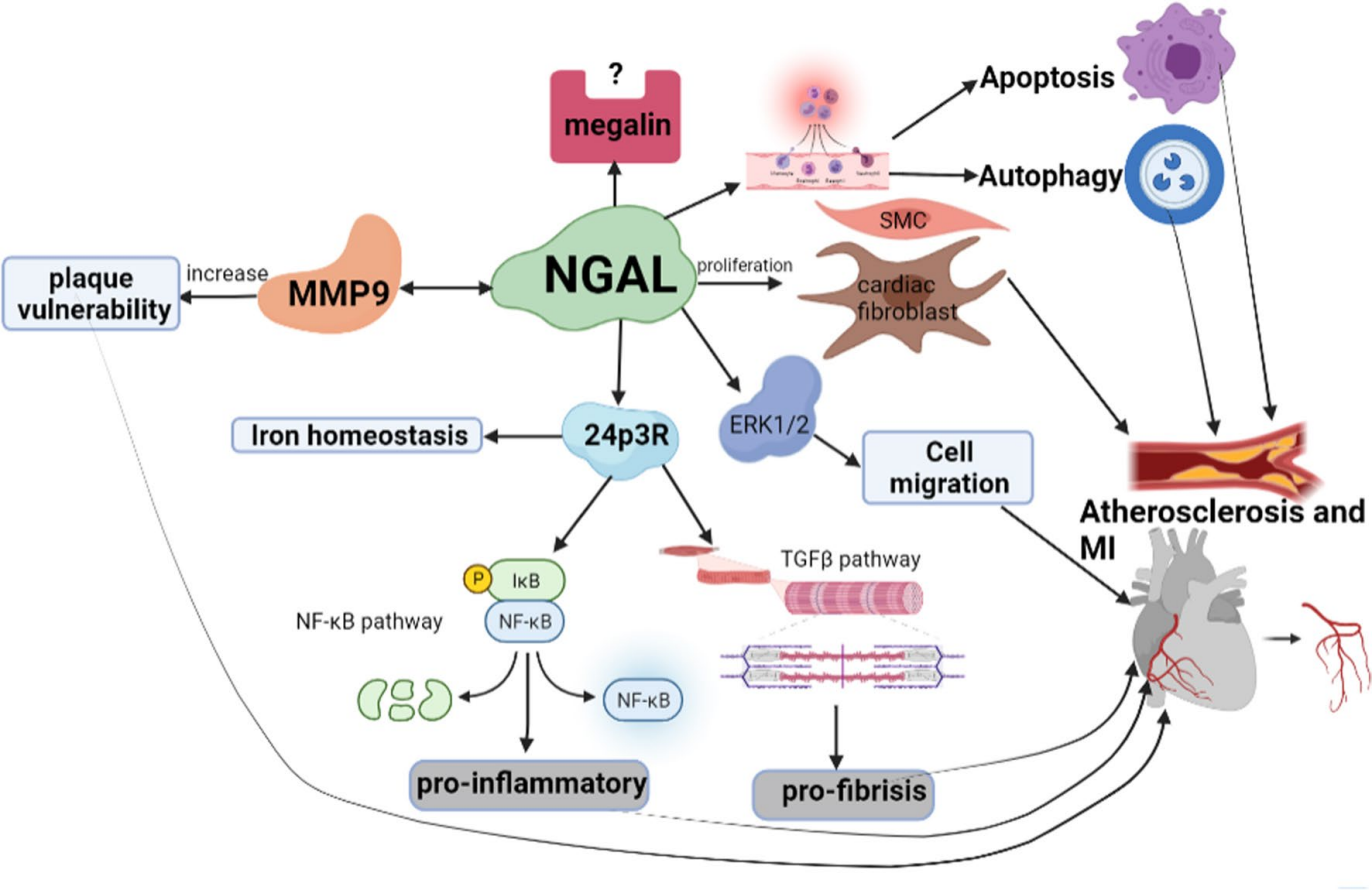
The roles and mechanisms of NGAL in ischemic heart disease. NGAL interacted with MMP-9 to increase plaque vulnerability. NGAL has two receptors, the 24p3R, and the megalin receptor. NGAL participates in controlling iron homeostasis, the activation of pro-inflammatory and pro-fibrosis signaling pathways through 24p3R. However, the role of the NGAL-megalin complex has not been well described. NGAL induced the proliferation of human vascular SMC and cardiac fibroblasts, which promoted the development of atherosclerosis and thus the occurrence of myocardial infarction. In addition, NGAL participates in ischemic heart disease by apoptosis, autophagy, and the Erk1/2 pathway. Abridgment NGAL is short for Neutrophil gelatinase-associated lipocalin; MMP-9 is short for matrix metalloproteinases 9; 24p3R is short for 24p3 receptor; ERK1/2 is short for extracellular regulated protein kinases 1/2; NF-κB is short for nuclear factor kappa-B; IκB is short for Inhibitor kappa B; TGF-β is short for Transforming growth factor β; MI is short for myocardial infarction; SMC is short for the smooth muscle cell. The question mark (?) indicates that some discrepancy still exists

The cysteine residue at position 87 in NGAL contributed to forming a disulfide bond bridge between NGAL and MMP-9, which can protect MMP-9 from degradation and preserve MMP-9 enzymatic activity [78]. Stabilization and accumulation of MMP-9 prevented extracellular matrix components from degradation and exacerbated tissue remodeling [79]. NGAL has two receptors, the 24p3 receptor (24p3R, also called lipocalin-2) and the megalin receptor [74]. 24p3R possesses a strong affinity ability for NGAL and mediates NGAL entering cells to control iron homeostasis and modulate intracellular iron concentration [74]. 24p3R has been demonstrated to express in the entire heart and the surface of cardiomyocytes [80]. In rat experimental autoimmune myocarditis, the expression of 24p3R was significantly up-regulated more than 100-fold [80]. It was also markedly increased in human myocarditis compared with non-inflammatory failing hearts [80]. 24p3R was expressed in pulmonary artery smooth muscle cells. Increased 24p3R expression was associated with over-activation of PI3K/Akt signaling resulted in promoting the proliferation of smooth muscle cells [81]. Aldosterone could significantly induce immune cell recruitment and NGAL expression in vivo experiments. NGAL associated 24p3R could promote and exacerbate aldosterone-induced cardiac remodeling and inflammation [82]. According to these published studies, AGAL might bind to 24p3R to mediate smooth muscle cells, promote inflammatory response and enhance aldosterone associated fibrosis in conditions of I/R or MI. However, it remains more experiments to investigate this deduction. The other known receptor of NGAL is megalin as well as called low-density lipoprotein receptor-related protein 2. Megalin has also been demonstrated to express in cultured cardiomyocytes and in immune cells, such as T cells, B cells, monocytes/macrophages, and granulocytes [83, 84]. However, the roles of the NGAL-megalin complex have not been well described in inflammation or ischemia-associated heart diseases (Fig. 2).

### The diagnostic and prognostic value of NGAL

Plasma NGAL levels are significantly higher in STEMI patients than in the stable angina pectoris patients and control subjects [85]. Multivariate regression analysis presented that NGAL levels were independently correlated to SYNTAX scores [85]. Plasma NGAL showed a better ability in discriminating severe coronary disease than MMP-9, hs-CRP, and IL-1β [85]. Plasma NGAL levels were markedly higher in death patients with STEMI than survivors. The ROC curve analysis showed that NGAL of more than 190 ng/mL could detect cardiovascular mortality in STMI patients with a sensitivity of 86% and specificity of 77% [86]. Among 260 STEMI patients admitted within 24 h after onsetting clinical symptoms, plasm NGAL on day 12 could predict combined adverse outcomes, including recurrent myocardial infarction, post-infarction angina, acute cerebrovascular accident, and death [87]. In another study that included 357 consecutive patients admitted to the hospital within 24 h after onseting STEMI, plasm NGAL level of more than 1.25 ng/mL on the 12th–14th day was associated with a 2.9-fold higher risk of a combined endpoint of cardiovascular death or any cardiovascular complication after 3 years of follow-up [88]. In a cohort of 119 post-MI patients who successfully accepted reperfusion after a first acute STEMI, higher baseline NGAL and a more significant increase in serum NGAL level indicated lower 6-month LV ejection fraction recovery evaluated by cardiac magnetic resonance [76]. Besides these short-term prognostic values of NGAL in acute coronary syndrome (ACS), plasm NGAL concentration has also been demonstrated to have a long-term predictive value in ACS. In a study of 1121 consecutive ACS patients with a follow-up of a median of 167 months, NGAL concentration could predict long-term mortality [89]. Plasm NGAL concentration above 2.6 ng/ml on day 12 after onsetting STMI was related to a fourfold increase of all-cause mortality during 3-year follow-up [90]. In a meta-analysis of 2462 STEMI patients with a mean follow-up duration of 13.9 years, plasm NGAL could forecast all-cause mortality and major adverse cardiovascular events (MACEs). STEMI patients in the higher NGAL group presented an approximately 47% and 52% greater risk of all-cause mortality and MACEs, respectively [91]. These studies implied that NGAL might be a potential and effective biomarker to discriminate risk stratification in coronary heart disease patients.

### NGAL’s treatment value

NGAL knockout mice exhibited lower LV interstitial fibrosis and inflammation, higher LV contractility and compliance, and greater stroke volume and cardiac output at 3 months post-MI than that in wild-type mice [76]. In another MI mouse model, NGAL knockout could effectively protect mouse heart from ischemia-induced cardiac functional abnormalities by inhibiting hypoxia-induced cytochrome C release and caspase-3 activation [92]. In a mouse I/R injury heart model of 20-min global ischemia followed by 60-min reperfusion, NGAL knockout mice exhibited significantly improved cardiac function and reduced infarct size compared to WT mice [93]. In the early stage of mouse cardiac allograft, I/R-induced significant up-regulation of NGAL and inflammatory cells infiltration; however, NGAL knockout could reduce approximately 54% granulocytes infiltration [94]. These studies demonstrated that inhibiting NGAL might be a potential strategy for treating myocardial ischemia–reperfusion injuries. However, more well-designed prospective studies are necessary to confirm the clinical value of NGAL inhibition.

## Cathelicidin

Cathelicidin (CRAMP in mouse/rat, LL-37 in humans) is an evolutionarily conserved component of the innate immune system that protects the host from various pathogens invasion. It is a natural broad-spectrum antibiotic and plays an essential role in regulating host defense and immunity [95, 96]. This peptide can be locally generated to a high level in the sites of inflammation and infection, which is produced mainly by many immune cells [97]. Cathelicidin is also expressed in epithelial cells of the intestine, airway, skin, and urinary tract, and germ cells. Cathelicidin has been discovered to neutralize lipopolysaccharide (LPS) and activate a wide range of receptors, including formyl-peptide receptor-like 1 (FPRL1), chemokine (C-X-C motif) receptor 2, and P2X purinoreceptor 7 (P2X7R), to mediate its multifunctional immunomodulatory activities. The non-bactericidal activities of cathelicidin, such as chemical attraction, immune cell activation, and angiogenesis, have attracted increasing attention [98, 99]. Recent studies have reported cathelicidins involved in cardiovascular diseases [97]. According to several studies, Cathelicidin is thought to have a role in the formation of atherosclerosis by activating platelets, recruiting inflammatory monocytes, and serving as a self-antigen. In LPS-induced endotoxemia model mice, cathelicidin deficiency exacerbated cardiac dysfunction [100]. In MI/R injuries, the role of cathelicidin remains unclear.

### The roles and mechanisms of cathelicidin in ischemia associated cardiac diseases

CRAMP was significantly down-regulated in both heart and cells from I/R mice and oxygen glucose-treated cardiomyocytes [97]. CRAMP (LL-37) was also significantly reduced in MI patients [97]. Knockdown of CRAMP in cardiomyocytes resulted in enhanced cellular apoptosis, and CRAMP deletion in mice displayed increased infarct size and myocardial apoptosis. Mechanistically, CRAMP peptide could activate phosphorylation of Akt and ERK1/2 and enhance the nuclear export of FoxO3a [97]. Mice subjected to MI surgery exhibited smaller scars, increased cardiac recovery, and decreased adverse remodeling after treating bone marrow mononuclear cells (BMMNCs) pre-incubated with CRAMP or injecting with hydrogels for sustained CRAMP release [101]. However, we could note that some other studies have supported that CRAMP might be detrimental in ischemia-associated cardiovascular disease. CRAMP administration in WT mice subjected to I/R increased myocardial inflammation, infarct size, and circulating cTnI, which could be effectively inhibited in CRAMP knockout I/R mouse models [102]. CRAMP administration mediated exacerbated mouse heart injury might be associated with enhancing TLR4 and P2X7R/NLRP3 signaling, since CRAMP administration mediated detrimental effects could be entirely reversed by inhibition of TLR4, P2X7R, and NLRP3 inflammasome [102]. Accordingly, it remains controversial whether CRAMP exerts a beneficial or a detrimental role in ischemia associated cardiovascular diseases, which need to be clarified in future studies.

## MMP-8

Matrix metalloproteinases (MMPs) are proteolytic enzymes that decompose extracellular matrix (ECM) components. Researches have reported that MMPs play a pivotal role in myocardial remodeling after MI. During the early stage after MI onset, cardiomyocyte necrosis led to activation of MMPs, which keep inactive proenzymes in the normal physiological condition. The overproduction and accumulation of MMPs cause imbalance expression between MMPs and tissue inhibitors of metalloproteinases, which contributes to the development of acute heart failure and acute aneurysm in the acute stage and progress of malignant cardiac remodeling and heart failure in the post-MI period. MMPs are composed of five subgroups according to their localization and substrate specificity containing collagenases, gelatinases, stromelysins, matrilysins, membrane-type MMPs, enamelysin, and others [103, 104]. MMP-8, also known as collagenase-2 or neutrophil collagenase, is mainly generated by neutrophils and macrophages [105].

### The roles and mechanisms of MMP-8 in ischemic heart disease

MMP-8 has been demonstrated to be secreted from neutrophil precursors during late myeloid maturation and fibroblasts, endothelial cells, smooth muscle cells. It usually keeps in an inactive pro-enzyme without biological functions under normal physiological conditions. However, MMP-8 could be converted into an active form after encountering a wide range of inflammatory stimuli and reactive oxygen species [106]. Once activated, MMP-8 could efficiently degrade collagen I, II, and III, which is very important for cardiac repairment after ischemic insults. Studies have exhibited that increased MMP expression is significantly associated with a cardiac remodeling in the human left ventricle of explanted hearts with ischemic dilated cardiomyopathy and in the myocardium with experimental MI [106, 107].

**Fig. 3:**
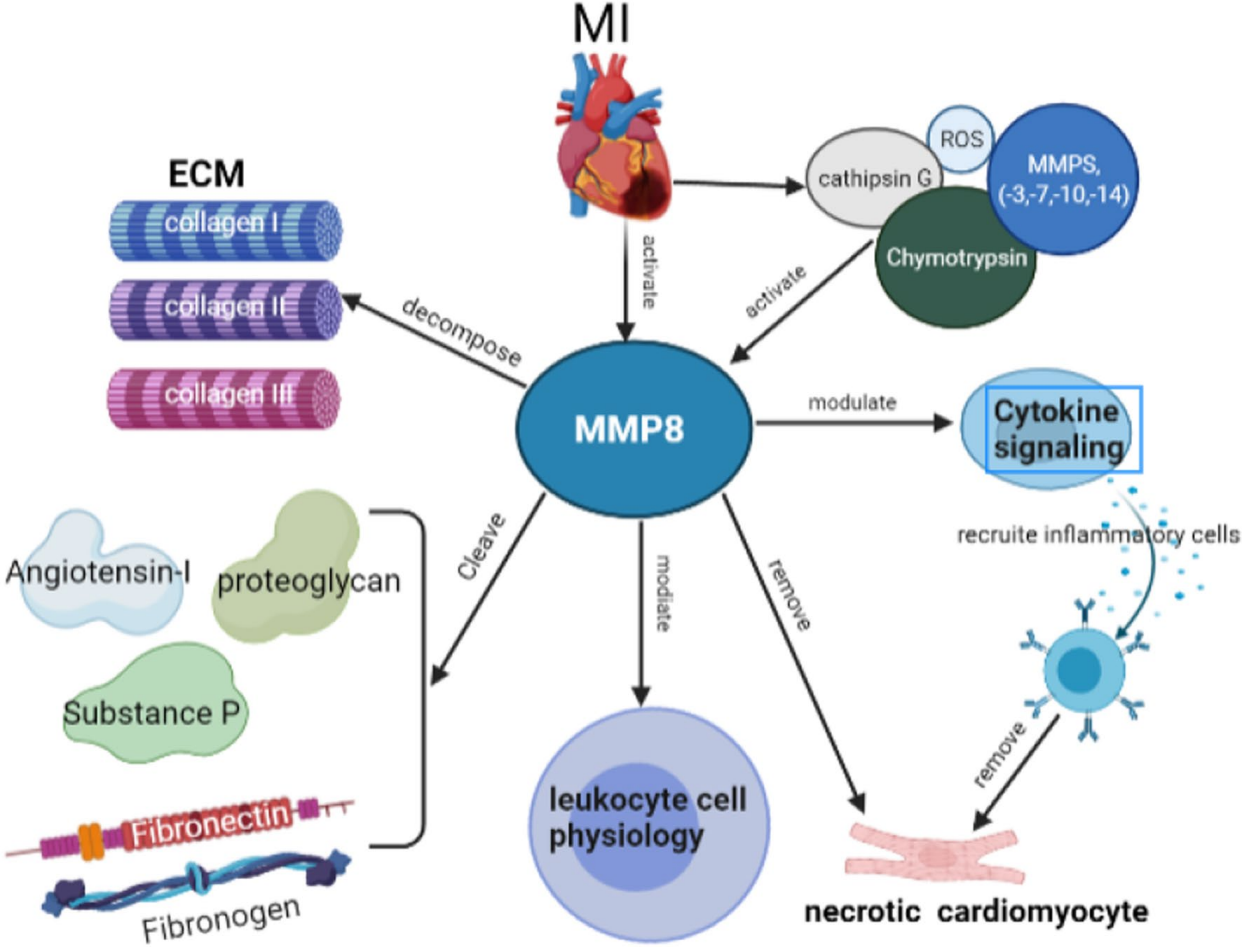
The roles and mechanisms of MMP-8 in ischemic heart disease. MMP-8 levels were increased in MI patients. The activation of MMP-8 is mediated by ROS or a variety of proteases like cathepsin G, chymotrypsin, or MMPs (-3, -7, -10, and -14). Activated MMP-8 cleaves a variety of proteins, among which the most famous substrates are type I, II, and III collagen. MMP-8 also cleaves various other proteins, including proteoglycan, fibronectin, fibrinogen, angiotensin-I, and substance P. In addition, MMP-8 modulated cytokine signaling, mediated leukocyte cell physiology, and recruited inflammatory cells to remove necrotic cardiomyocytes. *MMP-8* matrix metalloproteinases 8, *MI* myocardial infarction, *ECM* extracellular matrix, *ROS* reactive oxygen species

Fertin et al. examined the expression of MMP-1, -2, -3, -8, -9, -13, and TIMP-1, -2, -3, -4 in serum samples collected from MI patients at the time of discharge hospital, 1 month, 3 months and 1 year respectively [104]. Their study suggested that MMP8 and MMP9 have a significant positive correlation with malignant cardiac remodeling and left end-diastolic volume post-MI [104]. This association remained to be significant after adjusting for a series of covariates with MMP8 but without MMP9. MMP-2, -8, and 9 also have a significant positive correlation with cardiovascular death or hospitalization for heart failure during a 3-year follow-up. However, only MMP8 presented a significant association with adverse cardiovascular death or hospitalization [104]. In another study, the plasma MMP-8 expression remained higher in MI patients during 20 ± 3 months follow-up [108]. The up-regulated MMP-8 levels were positively associated with LVEF, end-diastolic volume, and end-systolic volume [108]. These studies suggested that plasma MMP-8 levels might be a potential biomarker for detecting and prognosis malignant cardiac remodeling in a long-term follow-up after MI (Fig. 3).

## MMP-9

MMP-9, located in tertiary granules, is one of the most extensively studied members of the MMP family in myocardial infarction. Neutrophils and macrophages are the early primary sources of MMP-9 after myocardial infarction. In addition, fibroblasts, cardiomyocytes, and endothelial cells can also secrete MMP-9 [109]. The inactive form of MMP-9, consisting of a pre-NH2-terminal domain, a conserved catalytic domain, a ligation domain, and a COOH-terminal blood-like catalytic lysis domain, is mainly stored in neutrophil gelatinase granules and released into the extracellular space after being stimulated by the phorbol ester formyl-Met-Leu-Phe, TNF-α, and IL-8 [110]. The proteolysis of proto-domains by other proteases such as MMP-1, -2, -3, -7, or -13 are mainly responsible for MMP-9 activation [111]. MMP-9 can also be activated by post-translational modifications of the domain cysteine residues, including S-glutathionylation or S-nitrosylation [112]. Once activated, MMP-9 could act on and dissolve a wide range of MI-associated substrates, including ECM proteins (mainly collagen, fibronectin, laminin, thrombo-reactive protein, and tendon in C), non-ECM substrates (mainly various cytokines and chemokines, such as TNFα, IL-1β, TGFβ, and CXC motif ligands), and novel substrates (CD36 and citrate synthase) [109].

### The roles and mechanisms of MMP-9 in ischemic heart disease

MMP-9 is mainly stored in gelatinase granules and subsequently released by inflammation or tissue damage associated with stimulation. So, neutrophils are a prominent and early source of MMP-9 in ischemic heart disease. Infiltrating neutrophils during the first hours of reperfusion of MI produced activated MMP9 [113]. After 30 min ischemia followed by 24 h reperfusion, the infarct area in the left ventricle of mouse hearts was decreased by 17.5% in MMP-9 heterozygotes and by 35.4% in MMP-9 knockout mice compared to wild type mice, respectively [114]. Plasma MMP-9 was positively associated with post-MI mortality and left ventricular dilation in the mouse-MI model. MMP-9 knockout enhanced expression of seven anti-inflammatory genes (CCL1, CCL6, CCR1, IL11, IL1r2, IL8rB, and Mif) and promoted anti-inflammatory polarization of macrophages without affecting pro-inflammatory polarization of macrophages in post-MI [115]. MMP-9 expression was also tested in a prospective cohort study with 91 acute myocardial infarction patients at intervals (0–12, 12–24, 24–48, 48–72, 72–96, and > 96 h). MMP9 reached a peak at 0–12 h and then kept up-regulation compared to the control group, followed by a fall to a plateau [116]. The higher early level of MMP9 was associated with worsened left remodeling and higher circulating white blood cells [116]. In other words, inhibiting MMP-9 expression at the early stage of MI might alleviate inflammatory response and attenuate cardiac malignant remodeling.

**Fig. 4:**
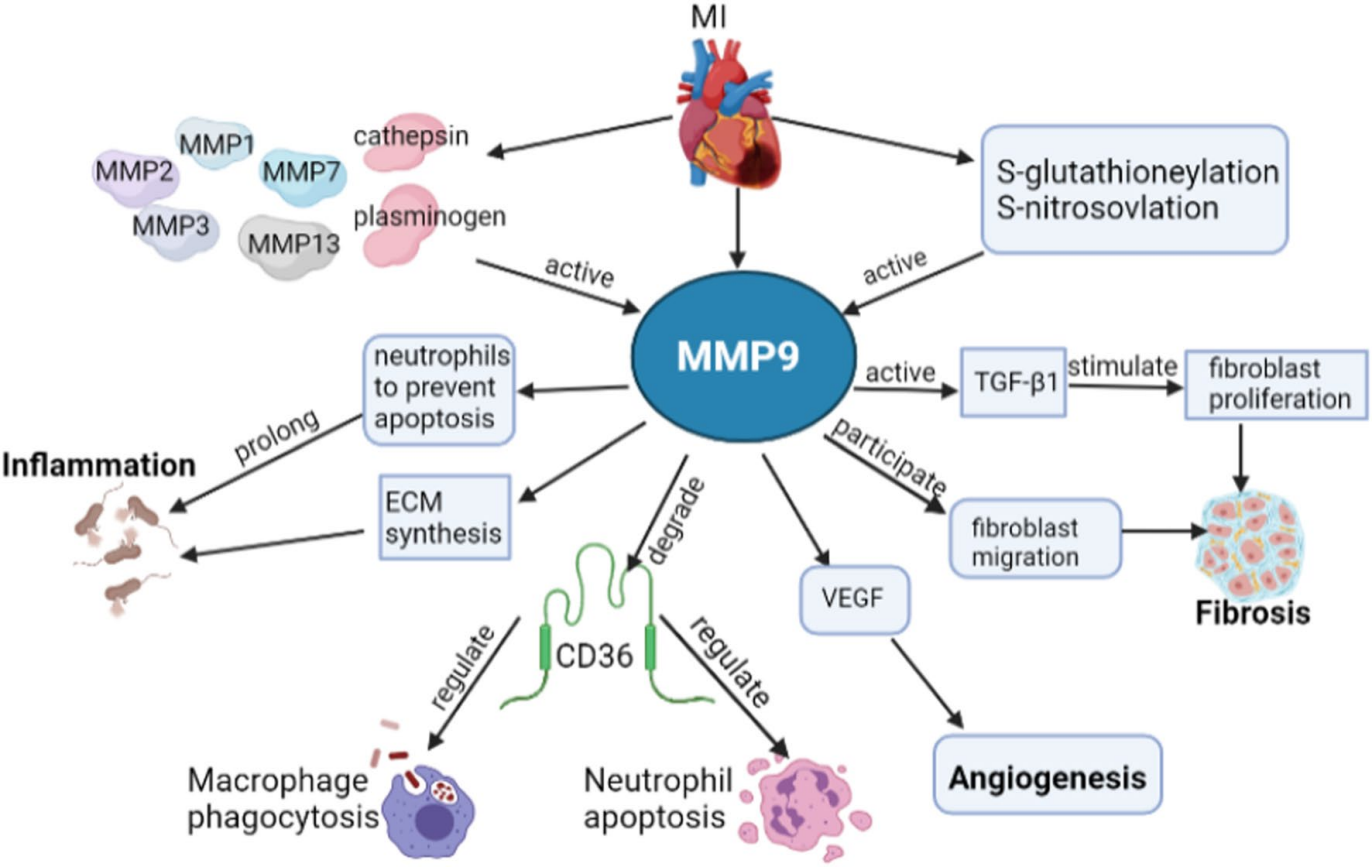
The mechanisms of MMP-9 in ischemic heart disease. The most common pathway for MMP-9 activation is hydrolytic proto-domains of other proteases such as MMP1, 2, 3, 7, or 13, cathepsin, and plasminogen. MMP-9 can also be activated by post-translational modifications of the domain cysteine residues, including S-glutathionylation or S-nitrosylation. The substrates of MMP-9 include ECM proteins (e.g. collagen, fibronectin, laminin, thrombo-reactive protein, and tendon in C), non-ECM substrates (various cytokines and chemokines, such as TNFα, IL-1β, TGFβ, and CXC motif ligands), and novel substrates (e.g. CD36 and citrate synthase). Thus, MMP-9 plays an essential role in ischemic heart disease by regulating macrophage phagocytosis, neutrophil apoptosis, inflammation, fibrosis, and angiogenesis. *MMP* matrix metalloproteinases, *MI* myocardial infarction, *ECM* extracellular matrix, *TGF-β* transforming growth factor β, *VEGF* vascular endothelial growth factor

Macrophages have been suggested to be another source of MMP-9. Kiugel et al. [117] demonstrated that MMP-9 expressed in macrophages and endothelial cells both at 7 days and 4 weeks after MI in rat myocardium by using 68 Ga-DOTA-peptide targeting MMP-2/9. However, transgenic overexpression of MMP-9 specifically in macrophages could significantly restrict extracellular matrix synthesis and attenuate MI-induced left ventricular function [118]. Mechanistically, MMP-9 transgenic overexpression decreased inflammatory markers expression both in macrophages isolated from MI mouse hearts and LPS treated mouse peritoneal macrophages [118]. In a clinical study with a small sample, Selejan et al. [119] showed that serum MMP-9 activity was significantly enhanced in acute myocardial infarction (AMI); however, the MMP-9 activity was highly depressed in AMI patients with cardiogenic shock. Mechanistically, activated MMP-9 could promote the production of soluble receptors for advanced glycation end products (s RAGE), limiting deleterious inflammation in cardiogenic shock. So, maintaining higher MMP-9 activity seemed to be a potential strategy to reduce AMI-associated cardiogenic shock [119]. These studies seemed to imply that different sources of MMP-9 play different roles in ischemic heart disease. This contradictory conclusion also indicated that we remained not fully understand the complexity of MMP-9 mechanisms of action in ischemic heart disease (Fig. 4).

### The prognostic value of MMP-9

In the culprit coronary of patients with STEMI, MMP-9 was significantly up-regulated compared to that in non-STEMI and stable angina patients [120]. MMP9 was further increased in the culprit coronary of posts-tent local [120]. This study exhibited that the MMP-9 expression might indicate the early clinical presentation in STEMI patients. Chen et al. [121] demonstrated that the expression of MMP-9 in peripheral blood of patients with STEMI was significantly up-regulated. MMP-9 could discriminate AMI patients from healthy subjects with a mean area under the receiver operating characteristic (ROC) curves of 0.81 and with diagnostic cut-off points of 690.066 ng/mL [122]. MMP-9 polymorphism might involve in AMI onset [123]. Serum MMP-9 expression was significantly up-regulated in AMI patients and was more associated with TT genotype in a clinical study containing 184 patients and 180 control subjects [123]. The MMP-9-1562T allele was more frequent in patients with AMI than in control subjects. The frequency of CT + TT genotypes seemed to be significantly associated with morbidity and mortality in patients with AMI than in control subjects [123]. These studies demonstrated that MMP-9 expression and serum level might be used as clinical biomarkers for predicting AMI. However, it needs more randomized controlled trials with a large sample size to further examine its predictive effects and ascertain the diagnostic cut-off points.

### The treatment value of MMP-9

In SPF SD rats, neuregulin-1 attenuated MI-induced dysfunctional cardiac electrical conduction by downregulating MMP-9 and upregulating Cx43 [124]. Apigenin ameliorated acute myocardial infarction in rats via inhibiting MMP-9 and inflammatory reactions [125]. Besides, trimetazidine suppressed oxidative stress, inhibited MMP-2 and MMP-9 expression, and prevented cardiac rupture in mice with MI [126]. A recent study found that Icariin attenuated myocardial apoptosis following myocardial infarction by inhibiting apoptosis and CD147/MMP-9 pathway [127]. In addition, salvianolic acid A, a novel MMP-9 inhibitor, is widely used to treat hypertension, coronary artery disease, and myocardial infarction [128]. However, effective MMP-9 inhibitors are far from development and therefore deserve further research.

## Arginase

Arginase (ARG) is mainly expressed in endothelial cells, red blood cells, and neutrophils [129, 130]. ARG has two isoforms, including ARG1 and ARG2 [131]. Although ARG1 and ARG2 presented only 58% homology in their structure, they showed the same active site [130, 131]. Thus, they have the same metabolites and exhibit similar biological activities [130, 131]. Arginase mainly hydrolyses L-arginine to urea and L-ornithine and regulates nitric oxide (NO) bioavailability by competing with NO synthase for their common substrate L-arginine. Increased arginase expression and activity contributed to the reduced availability of L-arginine. Arginase-mediated L-arginine exhaustion directly leads to decrease NO production but increased production of superoxide and accumulation of peroxynitrite. NO deficiency could inhibit vasodilators, promote cellular apoptosis and enhance the adhesion of neutrophils to vascular endothelium [132]. Accumulation of peroxynitrite and superoxide could directly cause cardiomyocytes' oxidative stress injuries. It has been suggested that the expression and activity of arginase were markedly up-regulated in the context of oxidative stress, atherosclerosis, hypertension, and ischemic heart disease. This review mainly summarizes the roles and mechanisms of arginase derived from neutrophils in ischemic heart disease.

### The roles and mechanisms of ARG1 in ischemic heart disease

Arginase 1 was significantly up-regulated in Sprague–Dawley rats subjected to 30 min of coronary artery ligation followed by 2 h of reperfusion [133]. Increased arginase competes with NOS for arginine utilization resulted in decreased NO production and citrulline/ornithine ratio [49, 133]. However, the arginase inhibitor N-omega-hydroxy-nor-L-arginine (nor-NOHA) treatment could increase tenfold of the citrulline/ornithine ratio and decrease the infarct size from 79 ± 4% to 39 ± 7% [133]. In another rat model subjected to 30 min coronary artery ligation and reperfusion up to 8 days, arginase activity was demonstrated to be markedly up-regulated as early as 20 min of reperfusion and maintained at 8 days [134]. Nor-NOHA treatment for arginase activity inhibition significantly reduced the area at risk at 2 h and 8 days of reperfusion, respectively [134]. The coronary flow velocity was increased dramatically during reperfusion in the nor-NOHA treatment group, which was inversely correlated with infarct size [134]. Arginase activity raised twofold in pig hearts subjected to coronary artery occlusion for 40 min followed by 4 h reperfusion [135]. Intracoronary nor-NOHA treatment decreased infarct size from 80 ± 4% to 46 ± 5%. However, combination treatment of nor-NOHA and the NO synthase inhibitor N(G)-monomethyl-L-arginine (L-NMMA) abolished nor-NOHA mediated cardio-protective effect [135]. The adverse effects of arginase were not limited to direct competition with NOS for common substrates. The decreased availability of L-arginine induced by arginase caused NOS decoupling, a phenomenon characterized by NOS producing superoxide rather than NO. Thus, arginase inhibition weakened the decoupling of eNOS. Thereby preventing the formation of superoxide and subsequent peroxy nitrites [124]. Arginase concentrations have also been demonstrated to be significantly up-regulated in MI patients compared to that in controls [136]. The increased arginase in MI patients was markedly negatively associated with left ventricular ejection fraction [136]. Mechanistically, increased arginase consumed arginine resulted in decreased NO production and increased oxidative stress [136].

### Arginase’s treatment value

As described above, arginase expression and activity were significantly up-regulated in ischemic heart disease. The underlying mechanism might be associated to compete with NO synthase for metabolizing NO substrate L-arginine resulted in reduced NO production and endothelial dysfunction. Thus, arginase inhibitors could restore NO production via enhancing L-arginine supply and eNOS activity. Several arginase inhibitors, including 2(S)-amino-6-boronohexanoic acid (ABH), nor-HOHA, and R-(2-boronoethyl)-L-cysteine (BEC), have been demonstrated to inhibit the arginase activity in vitro experiments and animal models. In a small clinical design including 16 patients with coronary artery disease (CAD), 16 patients with CAD and type 2 diabetes mellitus (CAD + DM), and 16 age-matched control subjects, intral-arterial infusion of nor-NOHA could significantly promote vasodilation as high as twofold in the CAD group and CAD + DM group but not in control subjects [137]. Moreover, Nor-NOHA showed significantly decreased infarct size after intracoronary treatment [135]; however, nor-NOHA presented no significant effect for reducing infarct size after intravenous treatment [135]. This might be explained by the rapid elimination (the mean residence time was 12.5 min) and high clearance owing to hydroxyguanidine chemical and metabolic lability [138]. Researches have been investigating new arginase inhibitors with characteristics of low clearance, long t1/2, and moderate volume distribution [139]. In summary, it is very promising that arginase inhibitor might improve endothelium function and attenuate ischemic heart diseases, but it still needs to be further verified by clinical studies (Tables 2 and 3).

**Table 2:** Diagnostic and prognostic value of granules in ischemic heart disease

Patients or experimental models	Major results	References
*MPO diagnostic value*		
Chest pain patients	1. MPO was a more efficient marker than CK-MB and cTn I within 0–6 h after the onset of AMI 2. A combination of MPO, CK-MB, and Tn I could discriminate 91% of the AMI patients as high as a specificity of 76%	[40]
MI patients	MPO as a valid test detection of MI yielded a specificity of 0.85	[41]
AMI patients	1. MPO levels increased in patients finally diagnosed with AMI even when Tn I exhibited a negative result at an early stage 2. MPO is more efficient than Tn I in AMI patients with a symptom onset of less than 2 h	[43]
Chest pain patients	Patients with a negative test by a higher sTn I assay, the value of MPO was most notable	[44]
Chest pain patients	1. MPO was inferior to the highly sensitive TnI in predicting AMI at 3 h and 6 h after admission of patients with chest pain 2. Both of the sensitivity and specificity were lower 3. MPO failed to provide incremental information when added to sTNI	[45]
*MPO prognosis value*		
ACS patients	MPO and Tn I were markedly associated with adverse cardiovascular events during hospitalization	[41]
MI patients	Higher MPO prospectively forecasts the outcome of MACE	[42]
ACS patients	MPO exhibited a strong prognosis value for MACE in serial sensitive cTnI negative patients	[44]
ACS patients	MPO was a predictive marker of increased risk of adverse events and mortality at 30 days and 6-month	[45]
AMI patients	Higher MPO predicted adverse cardiac outcome and lower ejection fraction	[46, 47]
AMI patients	MPO is a risk factor for long-term mortality	[48]
MI patients	MPO was an independent predictor of 6-month mortality and major adverse cardiac events	[49]
STEMI patients	Plasma MPO levels are correlated with plaque erosion	[50]
AMI patients	1. A high MPO level associated with more severe MO and IS 2. Higher MPO in the culprit artery indicated an exacerbated cardiac remodeling and infarct area at 6 months	[51]
ACS patients	1. Plasma MPO was significantly higher in STEMI patients than in NSTE-ACS patients 2. MPO failed to predict the short-term or long-term outcomes	[52]
*Azurocidin diagnostic value*		
STEMI patients	1. Azurocidin levels were significantly upregulated 2. Azurocidin was closely associated with thrombolysis 3. Azurocidin might be necessary for patients with STEMI	[71]
*NGAL diagnostic value*		
Post-MI patients	1. Plasma NGAL levels in STEMI patients were higher than those in the stable angina pectoris patients and control subjects 2. Plasma NGAL showed a better ability in discriminating severe coronary disease than MMP-9, hs-CRP, and IL-1β	[85]
MI patients	1. Plasma NGAL levels were markedly higher in death patients with STEMI than survivors 2. Plasma NGAL levels were increased in patients with acute and chronic heart failure as a complication of MI	[86]
*NGAL prognosis value*		
MI patients	Higher baseline NGAL and a more significant increase in serum NGAL level were correlated with lower 6-month LV ejection fraction recovery	[76]
AMI patients	1. Plasma NGAL level was significantly higher in death patients than in survived patients of AMI 2. Predict cardiovascular mortality in STEMI patients	[86]
STEMI patients	1. Plasm NGAL on day 12 could predict combined adverse outcomes 2. A marker of MI severity	[87]
STEMI patients	Plasm NGAL level of more than 1.25 ng/mL on the 12th–14th day was associated with a higher risk of a combined endpoint of cardiovascular death or any cardiovascular complication	[88]
ACS patients	NGAL concentration could predict long-term mortality	[89]
STEMI patients	Plasm NGAL level above 2.6 ng/ml on day 12 after onsetting STEMI was related to a fourfold increase of all-cause mortality	[90]
STEMI patients	STEMI patients in the higher NGAL group presented greater risk of MACEs and all-cause mortality	[91]
*Cathelicidin diagnostic value*		
Patients or MI mice	1. CRAMP was reduced from I/R mice and oxygen glucose treated cardiomyocytes 2. CRAMP was significantly reduced in MI patients	[97]
I/R mice	CRAMP might be detrimental in ischemia-associated cardiovascular disease	[102]
*MMP8 prognosis value*		
AMI patients	1. MMP-8 and MMP9 have a significant positive correlation with malignant cardiac remodeling and left end-diastolic volume post-MI 2. MMP8 presented a significant association with adverse cardiovascular death or hospitalization	[104]
AMI patients	The plasma MMP-8 level was still higher in MI patients during 20 ± 3 months follow-up	[108]
*MMP9 diagnostic value*		
MI patients	The higher early level of MMP9 was associated with worsened left remodeling	[116]
MI rats	1. MMP-9 accumulated in the damaged rat myocardium after an ischemic injury	[117]
MI mice	Transgenic overexpression of MMP-9 specifically in macrophages could significantly restrict extracellular matrix synthesis and attenuate MI-induced left ventricular function	[118]
AMI patients	1. MMP-9 serum activity is increased in AMI, but markedly suppressed in cardiogenic shock 2. Maintaining MMP-9 activity could be a therapeutic target to limit Receptor for advanced glycation end products-induced deleterious inflammation in cardiogenic shock	[119]
STEMI patients	The MMP-9 expression might indicate the early clinical presentation in STMI patients	[120]
STEMI patients	MMP-9 is considered a potential biomarker for the diagnosis of acute STEMI	[121]
AMI patients	MMP-9 could discriminate AMI patients from healthy subjects with a mean area under the receiver operating characteristic (ROC) curves of 0.81 and with diagnostic cut-off points of 690.066 ng/mL	[123]
AMI patients	The serum level of MMP-9 was associated with the risk of suffering AMI, and MMP-9 polymorphism and its level might be useful clinical biomarkers for predicting the outcome of AMI	[123]
*Arginase*		
MI patients	1. Arginase concentrations be significantly up-regulated in MI patients 2. The increased arginase in MI patients was markedly negatively associated with left ventricular ejection fraction	[136]

*MPO* myeloperoxidase, *pro-MPO* pro-myeloperoxidase, *CK* creatine kinase, *cTn I* troponin I, *STEMI* ST-segment elevation MI, *ACS* acute coronary syndromes, *sTn I* sensitive cardiac troponin I, *MO* microvascular obstruction, *IS* infarct size, *AKI* acute kidney injury, *MMPs* matrix metalloproteinases, *ROS* reactive oxygen species, *MACE* major adverse cardiovascular events

**Table 3:** Functional effects of targeting granules released by neutrophil degranulation in ischemic heart disease

Intervention	Research object	Age	Models	Drug dosage	Administration method	experimental period	Major outcome	Reference
PF-1355 (an oral MPO inhibitor)	Female C57BL/6J mice	8–12 weeks	MI	50 mg/kg of PF-1355 dissolved in vehicle excipient containing 40 mM Tris, 0.5% hydroxypropylmethylcellulose acetate succinate (HPMCAS) and 10% hydroxypropyl methylcellulose (HPMC), pH 10,	Twice daily by oral gavage	7 days	Decreased inflammation cells infiltration and attenuated left ventricular dilation	20
PF-1355 (an oral MPO inhibitor)	Female C57BL/6J mice	8–12 weeks	MI	50 mg/kg of PF-1355 dissolved in vehicle excipient containing 40 mM Tris, 0.5% hydroxypropylmethylcellulose acetate succinate (HPMCAS) and 10% hydroxypropyl methylcellulose (HPMC), pH 10	Twice daily by oral gavage	21 days of constant treatment	Both the cardiac function and remodeling were significantly improved	20
Pharmacological blockade of NE	Male C57BL/6 wild-type animal	Unknown	I/R	Unknown	Unknown	Unknown	Does not impact neutrophil transendothelial migration; Suppressed the increase in size of matrix protein low expression regions in the cremaster muscle I/R injury model	61
Sivelestat (an NE inhibitor)	C57BL/6J mice	Male approximately 10–12 weeks weighed at least 25 g	MI	100 mg/kg/day	Once daily by intraperitoneally injected	7 day	Improved survival and preserved cardiac function post-MI	62
Recombinant elafin (an endogenous neutrophil elastase inhibitor)	Patients	Perioperatively in patient	Patient undergoing coronary artery bypass surgery	200 mg intravenous bolus administered	EMPIRE Eudra CT 2010-019527-58	Unknown	Promising results (protective)	63
Sivelestat sodium hydrate (a selective NE inhibitor)	Swine	20–35 kg	Ligation of the left anterior descending coronary artery for 12-min, followed by 90-min reperfusion	6 and 60 mg/ml	Infused intracoronally	Starting just after reperfusion until the end of experiment	Attenuates myocardial contractile dysfunction due to myocardial stunning, thereby suppressing the production of interleukin-6 in activated neutrophils	64
Sivelestat (a NE inhibitor)	Adult male Wistar rats	Adult (240–300 g body weight)	I/R	Sivelestat was dissolved in KHB (10 μg/mL) to obtain a final concentration of 19 μmol/L	Infusion	10 min before ischemia and for the first 10 min of reperfusion	Attenuates myocardial injury after cardioplegic arrest	65
SSR69071 (an elastase inhibitor)	Male New Zealand white rabbits	Weighing 2–3 kg	Coronary artery occlusion for 30 min followed by reperfusion for 120 min	1 and 3 mg/kg	Intravenous intravenously	15 min before coronary ligation or 25 min after coronary ligation (5 min before reperfusion)	Reduces myocardial infarct size	66
The mCRAMP peptide	Male C57BL/6 mice	8–10 weeks	Ligation of the left anterior descending artery for 30 min followed by cardiac reperfusion for 24 h	4 mg/kg/day	Intraperitoneally injected	Three consecutive days	Inhibited cardiomyocyte apoptosis	97
The cathelicidin related antimicrobial peptide (CRAMP)	C57 BL/6 mice	Unknown	MI	10 μg/10 μL	Inject	5 weeks after treatment	Enhanced functional recovery, smaller scar size and higher capillary density	101
Neuregulin-1	Male Sprague Dawley rats	7–8 weeks old with average body weight 298.56 ± 38.73 g	AMI	10 μg/kg	Inject via the tail vein 2 h after the operation	Continued once daily for 7 days	Attenuates MI-induced dysfunctional cardiac electrical conduction	124
Apigenin	Male Wistar rats,	Weighing 220–250 g	AMI	10 mg/kg, 20 mg/kg and 40 mg/kg, respectively	Inject	Once a day	Ameliorates acute myocardial infarction of rats via inhibiting MMP-9 and inflammatory reactions	125
Trimetazidine	Male C57Bl/6 mice	Aged 8–12 weeks, weighing 22–25 g	MI	20 mg/kg/day	Intraperitoneal injection	7 days	Suppressed oxidative stress, inhibited MMP-2 and MMP-9, prevents cardiac rupture in mice with MI	126
Icariin	Male Sprague–Dawley rats	Age, 7–8 weeks Weight, 220–250 g	MI	At dosages of 3, 6, 12, and 20 mg/kg per day dissolved in the same amount of saline	Inject	28 days after surgery	Attenuated myocardial apoptosis following MI by inhibiting apoptosis and CD147/MMP-9 pathway	127
Arginase inhibitor N-omega-hydroxy-nor-L-arginine (nor-NOHA)	Male Sprague – Dawley rats	270– 400 g	30 min of coronary artery ligation, followed by 2 h of reperfusion	100 mg/kg	Intravenous as bolus injections	15 min before the onset of ischemia	Protects from MI. Increase tenfold of the citrulline/ornithine ratio and decrease the infarct size	133
Arginase inhibitor N(ω) -hydroxy-nor-l-arginine (nor-NOHA;	Male Wistar rats (Charles River, Germany)	Weight 300–350 g	30-min coronary artery ligation and reperfusion up to 8 days,	100 mg/kg	Intravenously	15 min before ischemia	Prevent the development of microvascular dysfunction and myocardial injury following I/R	134
Nrginase inhibitor (nor-NOHA	Female farm pigs	27–38 kg	Coronary artery occlusion for 40 min followed by 4 h reperfusion	2 mg/min	Systemic intravenous infusion	Started at 30 min of ischemia and continued up to 5 min after start of reperfusion	Local arginase inhibition during early reperfusion reduces infarct size	135

MPO: Myeloperoxidase; MI: myocardial infarction; NGAL: neutrophil gelatinase-associated lipocalin; IR: Ischemia–reperfusion; MMP: matrix metalloproteinases proteins; CRAMP: Cathelicidin; NE: neutrophil elastase. Nor-NOHA: N-hydroxy-nor-L-arginine

## Conclusion and outlook

Researches have reported that excessive neutrophil degranulation is a common characteristic of many inflammatory disorders [18, 140–142], including ischemic cardiomyopathy [14, 19, 22, 143]. Although regulating neutrophil degranulation might be an effective therapeutic strategy to attenuate a neutrophilic inflammatory response according to animal experiments and some small sample clinical studies, neutrophil degranulation remains to be clarified in the following aspects. Firstly, neutrophil degranulation goes through the early stagy of MI, the stage of high inflammatory response and sequentially inflammatory resolution, and the cardiac remodeling process after MI. It might be very different at the particle composition and cytokines secretion. A study with single-cell of transcriptomes and proteomics might be more beneficial to accurately analyze the particle composition of neutrophil degranulation and inflammatory cytokines’ distribution at different time points. Secondly, a series of inflammatory cytokines and secreted proteins were induced in the process of neutrophil degranulation. It is essential to define the roles and mechanisms of a single inflammatory cytokine or secreted protein via gene knockout or transgene strategy in future experiments. Thirdly, the signaling pathways involved in neutrophil degranulation remain to be elucidated in future experiments. Fourthly, the correlation and interaction between neutrophil degranulation and others such as macrophages and T cells remain to be clarified in future experiments. Fifthly, these secreted cytokines might not be derived only from neutrophil degranulation but also from other cells. For example, arginase could not only be secret from neutrophil degranulation but also highly expressed in endothelial cells. MMPs could not only be derived from neutrophil degranulation but also induced in cardiac fibroblast and macrophages. NGAL is not solely a product of neutrophil degranulation but also derives from activated macrophages. Thus, future studies should elucidate whether the same cytokines play different roles in different cells and diseases. In addition, some cytokines derived from neutrophil degranulation have been exhibited the diagnostic and prognostic value in the onset of MI and post-MI-associated cardiac malignant remodeling. However, the small sample and imperfect experimental designs limited its generalized application in clinical practice. Therefore, more studies and data are needed in future investigations by using long-term, large samples, and randomized control experiments.

## Data Availability

Not applicable.

## Abbreviations

IR: Ischemia–reperfusion
MI: Myocardial infarction
SMC: Smooth muscle cell
24p3R: 24P3 receptor
SOD2: Superoxide dismutase
Asn: Asparagines
Cys: Cysteine
MPO: Myeloperoxidase
HOCl: Hypochloric acid
Nrf2: NF-E2-related factor 2
I: Irisin
ULK1: Unc-51-like kinase-1
AMPK: 5’ AMP-activated protein kinase
eNOS: Endothelial nitric oxide synthase
NGAL: Neutrophil gelatinase-associated lipocalin
NLRP3: Nucleotide-binding oligomerization domain-like receptor protein 3
NF-κB: Nuclear factor kappa-B
ECM: Extracellular matrixc
ROS: Reactive oxygen species
IκB: Inhibitor kappa B
TGF-β: Transforming growth factor β
LDL: Low density lipoprotein
H_2_O_2_: Hydrogen peroxide
NO: Nitric oxide
HDL: High density lipoprotein
AKT: Protein kinase B
mTOR: Mammalian target of rapamycin
U: Uncoupling protein 2
VEGF: Vascular endothelial growth factor
MMPs: Matrix metalloproteinases proteins
ERK1/2: Extracellular regulated protein kinases 1/2
